# Impact of *ompk36* genotype and KPC subtype on the *in vitro* activity of ceftazidime/avibactam, imipenem/relebactam and meropenem/vaborbactam against KPC-producing *K. pneumoniae* clinical isolates

**DOI:** 10.1093/jacamr/dlad022

**Published:** 2023-03-23

**Authors:** Tara M Rogers, Ellen G Kline, Marissa P Griffith, Chelsea E Jones, Abigail M Rubio, Kevin M Squires, Ryan K Shields

**Affiliations:** School of Pharmacy, University of Pittsburgh, Pittsburgh, PA, USA; Department of Medicine, University of Pittsburgh, 3601 Fifth Avenue, Falk Medical Building, Suite 5B, Pittsburgh, PA 15213, USA; Department of Medicine, University of Pittsburgh, 3601 Fifth Avenue, Falk Medical Building, Suite 5B, Pittsburgh, PA 15213, USA; Department of Medicine, University of Pittsburgh, 3601 Fifth Avenue, Falk Medical Building, Suite 5B, Pittsburgh, PA 15213, USA; Department of Medicine, University of Pittsburgh, 3601 Fifth Avenue, Falk Medical Building, Suite 5B, Pittsburgh, PA 15213, USA; Department of Medicine, University of Pittsburgh, 3601 Fifth Avenue, Falk Medical Building, Suite 5B, Pittsburgh, PA 15213, USA; Department of Medicine, University of Pittsburgh, 3601 Fifth Avenue, Falk Medical Building, Suite 5B, Pittsburgh, PA 15213, USA; Center for Innovative Antimicrobial Therapy, University of Pittsburgh, Pittsburgh, PA, USA; Antibiotic Management Program, University of Pittsburgh Medical Center, Pittsburgh, PA, USA

## Abstract

**Objectives:**

The availability of new β-lactam/β-lactamase inhibitors ceftazidime/avibactam, meropenem/vaborbactam and imipenem/relebactam have redefined contemporary treatment of *Klebsiella pneumoniae* carbapenemase-producing *Klebsiella pneumoniae* (KPC-*Kp*) infections. We aimed to characterize and contrast the *in vitro* activity of these agents against genetically diverse KPC-*Kp* clinical isolates.

**Methods:**

We analysed genomes of 104 non-consecutive KPC-*Kp* isolates and compared the *in vitro* antibiotic activity by KPC subtype and *ompK36* genotype. MICs were determined in triplicate by CLSI methods. Twenty representative isolates were selected for time–kill analyses against physiological steady-state and trough concentrations, as well as 4× MIC for each agent.

**Results:**

Fifty-eight percent and 42% of isolates harboured KPC-2 and KPC-3, respectively. *OmpK36* mutations were more common among KPC-2- compared with KPC-3-producing *Kp* (*P *< 0.0001); mutations were classified as IS*5* insertion, glycine-aspartic acid insertion at position 134 (GD duplication) and other mutations. Compared to isolates with WT *ompK36*, ceftazidime/avibactam, imipenem/relebactam and meropenem/vaborbactam MICs were elevated for isolates with IS*5* by 2-, 4- and 16-fold, respectively (*P *< 0.05 for each). Against isolates with GD duplication, imipenem/relebactam and meropenem/vaborbactam MICs were increased, but ceftazidime/avibactam MICs were not. In time–kill studies, ceftazidime/avibactam-mediated killing correlated with ceftazidime/avibactam MICs, and did not vary across *ompK36* genotypes. Imipenem/relebactam was not bactericidal against any isolate at trough concentrations. At steady-state imipenem/relebactam concentrations, regrowth occurred more commonly for isolates with IS*5* mutations. Log-kills were lower in the presence of meropenem/vaborbactam for isolates with GD duplication compared with IS*5* mutations.

**Conclusions:**

Our investigation identified key genotypes that attenuate, to varying degrees, the *in vitro* activity for each of the new β-lactam/β-lactamase inhibitors. Additional studies are needed to translate the importance of these observations into clinical practice.

## Background

Infections due to carbapenem-resistant Enterobacterales (CRE) are a major cause of attributable death worldwide.^1^ In the USA alone, CRE infections are responsible for over 13 ,000 hospitalizations, 1,100 deaths, and $130 million in healthcare costs each year.^2^ The most common CRE pathogen in US-based surveillance studies is *Klebsiella pneumoniae*, the majority of which harbour *K. pneumoniae* carbapenemase (KPC) enzymes.^3,4^ Accordingly, KPC has been a primary target for drug discovery efforts, leading to the development of three novel β-lactamase inhibitors that inhibit KPC: avibactam, relebactam and vaborbactam. Avibactam and relebactam are diazabicyclooctane β-lactamase inhibitors that have been partnered with ceftazidime and imipenem, respectively. Vaborbactam is a cyclic boronic acid β-lactamase inhibitor paired with meropenem. The resulting combinations of ceftazidime/avibactam, imipenem/relebactam and meropenem/vaborbactam have each shown potent *in vitro* activity against KPC-producing *K. pneumoniae* (KPC-*Kp*),^5–7^ and favourable clinical outcomes for patients infected with carbapenem-resistant pathogens.^8–11^ Each are now recommended as preferred treatment options for the management of patients with CRE infections due to KPC-producing Enterobacterales.^12,13^ Which agent should be prioritized for KPC-*Kp* specifically, however, is still unclear.

Carbapenem resistance in KPC-*Kp* is predominantly mediated by KPC; however, the loss or modification of major porin channels in the outer cell membrane can contribute to high-level resistance.^14,15^ The two major porins in *K. pneumoniae* are OmpK35 and OmpK36, which are encoded by *ompK35* and *ompK36*, respectively. Loss-of-function mutations in *ompK35* are common among KPC-*Kp.*^7,16^*ompK36* genotypes, on the other hand, vary significantly among clinical isolates. Two major mutations have been described in KPC*-Kp.* The first in an IS*5* insertion element within the *ompK36* gene promoter or coding sequence that is associated with decreased *ompK36* expression.^14,17^ The second is a 6 bp insertion in the L3 loop encoding a glycine and aspartic acid. This insertion is known to constrict the inner pore diameter and subsequently limit intake of nutrients as well as antibiotics like the carbapenems.^15,16^ It is unclear if other reported mutations or insertions in *ompK36* impact the activity of carbapenems, or more specifically the activity of ceftazidime/avibactam, imipenem/relebactam or meropenem/vaborbactam.

The available data show that various molecular mechanisms can contribute to the *in vitro* activity of ceftazidime/avibactam, imipenem/relebactam and meropenem/vaborbactam. Translating these data to inform clinical decisions is a particular challenge because all three agents are likely to be reported as susceptible for the vast majority of KPC-*Kp*. We hypothesized that the underlying molecular mechanisms of resistance in KPC-*Kp* contribute to the *in vitro* activity of these agents to varying degrees. The objective of this study was to compare the *in vitro* activity and killing effects for ceftazidime/avibactam, meropenem/vaborbactam and imipenem/relebactam against genetically diverse KPC-*Kp* clinical isolates.

## Materials and methods

### Characterization of Kpc-*Kp* clinical isolates

Clinical isolates collected from patients not previously treated with ceftazidime/avibactam, imipenem/relebactam or meropenem/vaborbactam were selected from local biorepositories. All isolates were stored at −80°C and subcultured twice on Mueller–Hinton agar (MHA; Becton, Dickinson, & Company, Sparks, MD, USA) prior to testing. WGS was performed on an Illumina platform as described previously.^18^ Species, ST and KPC subtypes were confirmed with WGS analyses. Core-genome SNPs were identified using pairwise comparisons with Snippy (https://github.com/tseemann/snippy). Genotypes for *ompK35* were categorized as either WT or truncated. Genotypes for *ompK36* were denoted as WT or mutant. Mutant *ompK36* genotypes included IS*5* (IS*5* insertion element in either promoter region or *ompK36* coding sequence), GD duplication (glycine and aspartic acid insertions at amino acid positions 134 and 135) or other (one or more divergent sequences, insertions, substitutions or deletions) mutations. WT *ompK35* and *ompK36* genes were defined as described previously.^14,15,19^

### Susceptibility testing

MICs were determined in triplicate by standardized broth microdilution methods; susceptibility was defined according to CLSI interpretive criteria.^20^ Tested concentrations of ceftazidime/avibactam, imipenem/relebactam and meropenem/vaborbactam ranged from 0.12–256, 0.06–64 and 0.008–8 mg/L, respectively. β-Lactamase inhibitors avibactam, relebactam and vaborbactam were tested as fixed concentrations of 4, 4 and 8 mg/L, respectively. Quality control was assessed using *Pseudomonas aeruginosa* ATCC 27853 and *K. pneumoniae* ATCC 700603, and results were reported only when MICs for control strains were within acceptable ranges.

### In vitro killing activity

Time–kill assays were performed on 20 isolates that were representative of the predominant KPC subtypes and *ompK36* genotypes. Each isolate was grown overnight in CAMHB (Becton, Dickinson, & Company, Sparks, MD) at 37°C with shaking. Experiments were performed in 8 mL of CAMHB using an initial inoculum of 1 × 10^6^ cfu/mL. Ceftazidime/avibactam, imipenem/relebactam and meropenem/vaborbactam were tested at a concentration of 4× MIC. In addition, physiological free drug steady-state and trough concentrations were identified from published studies.^21–23^ Steady-state exposures included 32, 4 and 8 mg/L for ceftazidime/avibactam, imipenem/relebactam and meropenem/vaborbactam, respectively. Corresponding trough concentrations were 4, 0.5 and 1 mg/L, respectively. Simulated trough concentrations were selected to represent the lowest exposure achieved in patients prior to administration of the next dose. All experiments were incubated at 37°C with 200 rpm shaking. Samples were taken at 0, 2, 6, 10 and 24 h, serially diluted, plated on Mueller–Hinton agar plates, and incubated overnight at 37°C. Colonies were enumerated and reported as cfu/mL; bactericidal activity was defined as ≥3 log kill after 24 h of incubation compared with the starting inoculum.

### Statistical analysis

Categorical and continuous variables were analysed using a chi-squared (or Fisher’s exact) test and Mann–Whitney *U*-test, respectively. Mean log-kills after 24 h were compared using a Student’s *t*-test. A two-tailed *P* value of ≤ 0.05 was considered statistically significant.

## Results

**Figure 1.**
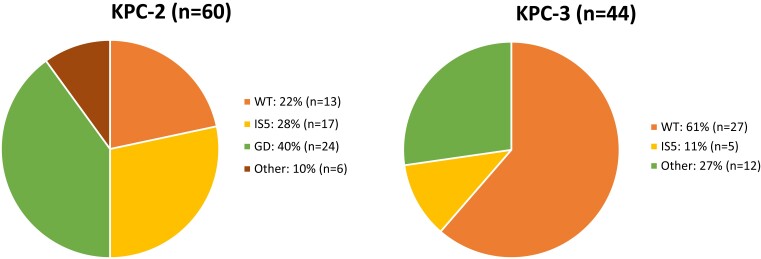
Distribution of *ompK36* genotypes stratified by KPC subtype. Mutations described as ‘other’ are reported in Table S1.

A total of 104 KPC-*Kp* isolates from unique patients were included in the study. Ten different STs were represented; ST258 accounted for 88% (92/104) of isolates (Table S1, available as Supplementary data at *JAC-AMR* Online). All isolates harboured KPC-2 (*n* = 60) or KPC-3 (*n* = 44), but not other KPC variants. Ninety-six percent (100/104) of isolates contained a truncated *ompK35* gene. Among *ompK36* genotypes, 38% (40/104) were WT and the remaining 62% (64/104) contained mutant genotypes. *ompK36* mutations were categorized as GD duplication (*n* = 24), IS*5* (*n* = 22) or other (*n* = 18), and were more common among KPC-2-producing isolates as compared with KPC-3-producing isolates (78% versus 39%; *P* < 0.0001; Figure 1).

**Figure 2.**
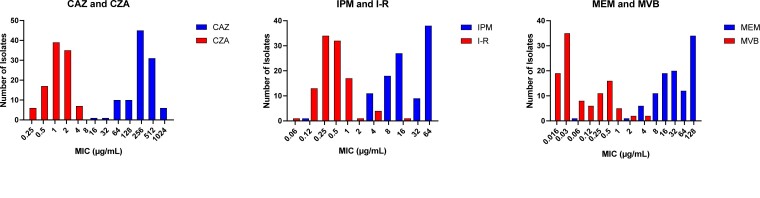
MIC distribution for each β-lactam and β-lactam/β-lactamase inhibitor agent tested against KPC-*Kp*. Median ceftazidime, imipenem and meropenem MICs were reduced by 256-, 32- and 512-fold with the addition of avibactam, relebactam and vaborbactam, respectively. CAZ, ceftazidime; CZA, ceftazidime/avibactam; IPM, imipenem; I-R, imipenem/relebactam; MEM, meropenem; MVB, meropenem/vaborbactam.

**Figure 3.**
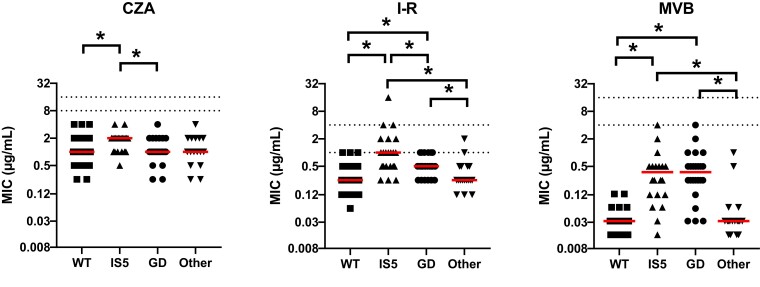
Comparison of ceftazidime/avibactam, imipenem/relebactam and meropenem/vaborbactam MICs stratified by *ompK36* genotype. Dotted lines represent CLSI susceptible and resistance breakpoints for each agent. Solid horizontal lines represent the median MIC for each group of isolates. * denotes *P* < 0.05. CZA, ceftazidime/avibactam; I-R, imipenem/relebactam; MVB, meropenem/vaborbactam.

**Figure 4.**
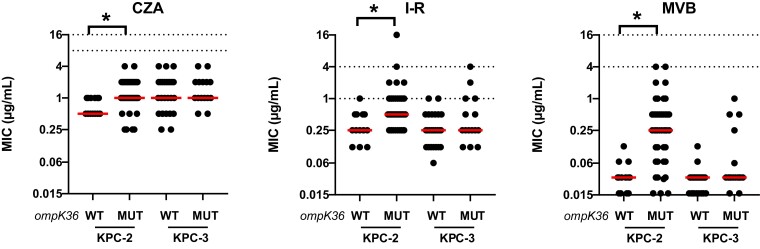
Comparison of ceftazidime/avibactam, imipenem/relebactam and meropenem/vaborbactam MICs stratified by KPC subtype and *ompK36* genotype. Dotted lines represent CLSI susceptible and resistance breakpoints for each agent. Horizontal lines represents the median for each group of isolates. * denotes *P* < 0.05. CZA, ceftazidime/avibactam; I-R, imipenem/relebactam; MUT, mutant *ompK36* genotype; MVB, meropenem/vaborbactam.

All isolates were non-susceptible to ceftazidime; 99% (103/104) were non-susceptible to each imipenem and meropenem. The addition of avibactam, relebactam and vaborbactam lowered median ceftazidime, imipenem and meropenem MICs by 256-, 32- and 512-fold, respectively (Figure 2). The resulting rates of susceptibility for ceftazidime/avibactam, imipenem/relebactam and meropenem/vaborbactam against KPC-*Kp* were 100%, 93% and 100%, respectively (Table 1). Median MICs did not vary by the presence or absence of truncated *ompK35* genes. Against isolates with WT *ompK36* genotypes (*n* = 42), median ceftazidime/avibactam MICs were higher against isolates with KPC-3 compared with KPC-2 (1 versus 0.5 mg/mL; *P = *0.02). Median imipenem/relebactam and meropenem/vaborbactam MICs did not differ by KPC subtype. By specific *ompK36* genotype (Figure 3), median MICs were higher for ceftazidime/avibactam, imipenem/relebactam and meropenem/vaborbactam against isolates with IS*5* compared with WT (2 versus 1 mg/L, *P = *0.003; 1 versus 0.25 mg/L, *P *< 0.001; and 0.375 versus 0.032 mg/L, *P < *0.001; respectively). Compared with WT, the corresponding median MICs among isolates with IS*5* genotypes were 2-, 4- and 16-fold higher, respectively. Against isolates with GD duplication, median imipenem/relebactam (2-fold; 0.5 versus 0.25 mg/L, *P* = 0.024) and meropenem/vaborbactam (16-fold; 0.375 versus 0.032 mg/L, *P *< 0.001) MICs were higher than WT, but median ceftazidime/avibactam MICs did not differ. Median MICs for isolates harbouring other *ompK36* mutations did not vary compared with WT for any of the three agents. When comparing both KPC subtype and *ompK36* genotypes, median MICs were increased against KPC-2-producing isolates with *ompK36* mutations, but not for KPC-3-producing isolates (Figure 4). In the presence of KPC-2, median MICs against WT compared to *ompK36* mutant isolates increased from 1 to 2 mg/L (*P = *0.0006), 0.25 to 0.5 mg/L (*P = *0.003) and 0.03 to 0.25 mg/L (*P *< 0.0001) for ceftazidime/avibactam, imipenem/relebactam and meropenem/vaborbactam, respectively.

**Table 1.** MICs of β-lactam and β-lactam/β-lactamase inhibitor agents against KPC-*Kp* clinical isolates

Drug	*ompK36* Classification (total number of isolates)	MIC_50_	MIC_90_	Range	% S	% I	% R
Ceftazidime	All (*n* = 104)	256	512	16 to ≥ 1024	0	0	100
Ceftazidime/avibactam	All (*n* = 104)	1	2	≤0.25–4	100	—	0
	WT (*n* = 40)	1	2	≤0.25–4	100	—	0
	IS*5* (*n* = 22)	2	2	0.5–4	100	—	0
	GD (*n* = 24)	1	2	≤0.25–4	100	—	0
	Other (*n* = 18)	1	2	≤0.25–4	100	—	0
Imipenem	All (*n* = 104)	16	64	0.12 to ≥ 64	1	0	99
Imipenem/relebactam	All (*n* = 104)	0.5	1	0.06–16	93	4	3
	WT (*n* = 40)	0.25	0.5	0.06–1	100	0	0
	IS*5* (*n* = 22)	1	4	0.25–16	72	14	14
	GD (*n* = 24)	0.5	1	0.25–1	100	0	0
	Other (*n* = 18)	0.25	0.5	0.12–2	94	6	0
Meropenem	All (*n* = 104)	32	128	0.06 to ≥ 128	1	1	98
Meropenem/vaborbactam	All (*n* = 104)	0.03	0.5	0.015–4	100	0	0
	WT (*n* = 40)	0.03	0.06	0.015–0.5	100	0	0
	IS*5* (*n* = 22)	0.5	1	0.015–4	100	0	0
	GD (*n* = 24)	0.5	1	0.03–4	100	0	0
	Other (*n* = 18)	0.03	0.06	0.015–1	100	0	0

Rates of susceptibility were interpreted according to CLSI breakpoints. MICs are expressed in mg/L. I, intermediate; R, resistant; S, susceptible.

Next, 20 isolates were selected for time–kill analyses; the median core-genome SNP difference across isolates was 76 (range: 2–18, 291). Isolates were selected to represent each of the four predominant KPC-*Kp* subgroups associated with MIC differences. Five isolates from each subgroup of KPC-2 with WT *ompK36*, KPC-3 with WT *ompK36*, KPC-2 with IS*5*, and KPC-2 with GD duplication were included. Corresponding within-group median core-genome SNP differences were 46, 128, 50 and 33, respectively (Table S2).

**Figure 5.**
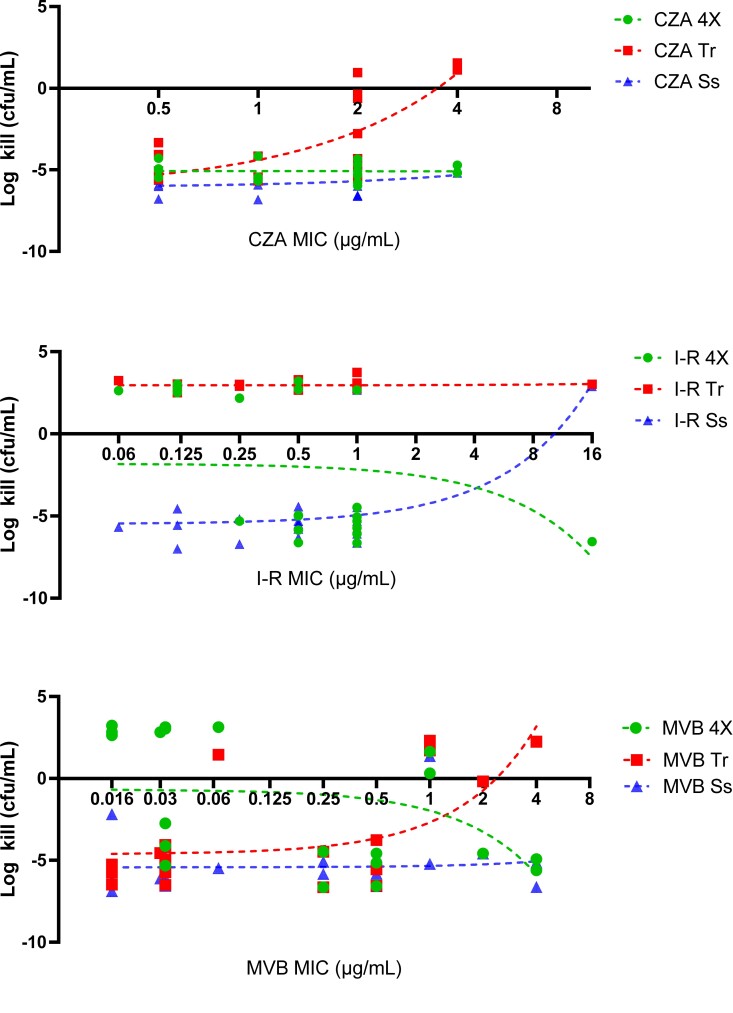
24 h log–kills across KPC-*Kp* stratified by ceftazidime/avibactam, imipenem/relebactam and meropenem/vaborbactam MICs. 24 h log-kills were calculated as the difference in cfu/mL at 0 and 24 h and plotted according to the baseline MIC for each isolate. Dotted lines indicate the best fit linear regression line. CZA, ceftazidime/avibactam; I-R, imipenem/relebactam; MVB, meropenem/vaborbactam; 4×, 4× MIC; Tr, simulated trough concentration; Ss, simulated steady-state concentration.

**Figure 6.**
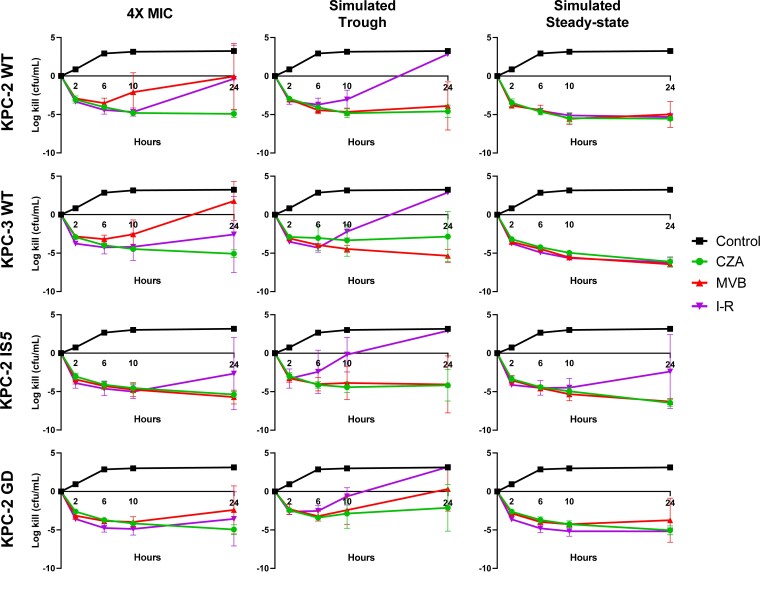
Mean log-kills across KPC-*Kp* exposed to various concentrations of ceftazidime/avibactam, imipenem/relebactam and meropenem/vaborbactam. Individual graphs demonstrate the mean log-kills at each time point over the 24 h incubation period. Error bars show the standard deviation. CZA, ceftazidime/avibactam; I-R, imipenem/relebactam; MVB, meropenem/vaborbactam; 4×, 4× MIC; Tr, simulated trough concentration; Ss,  simulated steady-state concentration.

Against physiological steady-state (32 mg/L) and trough (4 mg/L) concentrations, ceftazidime/avibactam was bactericidal against 100% and 70% of isolates, respectively. In the presence of ceftazidime/avibactam trough concentrations, mean log-kills were greater against isolates with ceftazidime/avibactam MICs ≤1 mg/L (−4.93 cfu/mL) compared with isolates with MICs ≥2 mg/L (−1.92 cfu/mL; *P = *0.004; Figure 5). At ceftazidime/avibactam exposures of 4× MIC, mean log-kills did not vary against isolates with WT (log-kill = −4.99 cfu/mL) or mutant (log-kill = −5.17 cfu/mL) *ompK36* genotypes (Figure 6).

Imipenem/relebactam was bactericidal against 90% of isolates at steady-state concentrations (4 mg/L), demonstrating mean log-kills of −4.77 cfu/mL across all isolates. In contrast, trough concentrations (0.5 mg/L) were not bactericidal against any isolate (Figure 5). In the presence of imipenem/relebactam concentrations of 4× MIC, mean log-kills against isolates with WT, IS*5* and GD duplication *ompK36* genotypes were −1.48, −2.69 and −3.57 cfu/mL, respectively. There was no difference in mean log-kills or rates of bactericidal activity for isolates with IS*5* or GD duplication genotypes; however, bacterial regrowth was noted for isolates with IS*5* mutations, but not GD duplication against steady-state concentrations (Figure 6). Paradoxically, log-kills with imipenem/relebactam at 4× MIC were greater against isolates with MICs ≥1 mg/L (−4.65 cfu/mL) compared with isolates with MICs ≤0.5 mg/L (−0.78 cfu/mL; *P = *0.04).

Meropenem/vaborbactam was bactericidal against 90% and 70% of isolates in the presence of steady-state (8 mg/L) and trough (1 mg/L) concentrations, respectively. Concentrations of 4× MIC were below simulated trough concentrations for all isolates with WT *ompK36*, resulting in lower mean log-kills in the presence of meropenem/vaborbactam at 4× MIC (0.86 cfu/mL) compared with trough concentrations (−4.61 cfu/mL; *P = *0.0006). For isolates with *ompK36* mutant genotypes, meropenem/vaborbactam 4× MIC resulted in greater killing against isolates with IS*5* mutations (−5.71 cfu/mL) compared with GD duplication (0.32 cfu/mL; *P = *0.002); regrowth after 10 h was observed against GD duplication, but not IS*5* genotypes.

## Discussion

In this study, we compared the MICs and *in vitro* killing activity of ceftazidime/avibactam, imipenem/relebactam and meropenem/vaborbactam against KPC-*Kp* clinical isolates with the most common KPC subtypes and *ompK36* genotypes encountered clinically. Our data demonstrate several key differences between the agents which may help differentiate front-line treatment options for KPC-*Kp* infections. First, we show that MICs for each agent were elevated against isolates harbouring IS*5* mutations in *ompK36.* Such mutations increased the median MICs (compared with WT) by 2-, 4- and 16-fold for ceftazidime/avibactam, imipenem/relebactam and meropenem/vaborbactam, respectively. While the magnitude of MIC change was greatest for meropenem/vaborbactam, rates of *in vitro* susceptibility did not change (Table 1). In contrast, 28% of KPC-*Kp* with IS*5* mutations in *ompK36* were categorized as non-susceptible to imipenem/relebactam. These findings align with attenuated killing and regrowth during time–kill studies in the presence of physiological trough and steady-state imipenem/relebactam concentrations against KPC-*Kp* with IS*5* mutations (Figure 6). We did not identify differences for the *in vitro* killing activity of ceftazidime/avibactam against isolates with or without IS*5* mutations, suggesting that this agent was the least impacted of the three agents tested. Median imipenem/relebactam and meropenem/vaborbactam, but not ceftazidime/avibactam, MICs were also increased against KPC-*Kp* harbouring a GD duplication in *ompK36* (Figure 3). At simulated steady-state concentrations, imipenem/relebactam and meropenem/vaborbactam were bactericidal against 100% and 80% of isolates harbouring GD duplications, respectively; however, at trough concentrations, corresponding rates of bactericidal killing were reduced to 0% and 20%, respectively. In fact, trough concentrations of imipenem/relebactam were not bactericidal against any KPC-*Kp* isolate studied. These data suggest that each major *ompK36* mutation has a differential impact on ceftazidime/avibactam, imipenem/relebactam and meropenem/vaborbactam. Other less common mutations in *ompK36* did not impact MICs overall; however, two isolates demonstrating elevated meropenem/vaborbactam and imipenem/relebactam MICs were noted. One isolate harboured a glycine to alanine substitution 11 bp upstream of *ompK36*, and the other showed an amino acid insertion at position 104 resulting in a frameshift (Table S1). Finally, we noted paradoxical killing effects with imipenem/relebactam and meropenem/vaborbactam concentrations at 4× MIC, such that log-kills were greater against isolates with elevated MICs (Figure 5). We anticipate that greater killing of isolates harbouring *ompK36* mutations was due to a potential fitness cost, or alternatively selection of a resistant subpopulation for isolates with WT genotypes at baseline.

Our findings corroborate and extend previous reports demonstrating the impact of KPC subtypes and *ompK36* mutations on the activity of ceftazidime/avibactam, imipenem/relebactam and meropenem/vaborbactam.^5–7,24^ We have identified key genotypes that attenuate, to varying degrees, the activity for each agent. For ceftazidime/avibactam, MICs are 2-fold higher for KPC-3 compared with KPC-2-producing isolates. At the same time, ceftazidime/avibactam was the least impacted of the three agents by mutations in *ompK36* where only IS*5* mutations resulted in a 2-fold MIC increase, but no change in the killing activity for any ceftazidime/avibactam concentration tested in time–kill studies.

Next, we found that imipenem/relebactam MICs increased in a stepwise manner across *ompK36* genotypes (Figure 3). Median MICs increased from 0.25 to 0.5 to 1 mg/L against KPC-*Kp* with *ompK36* WT, GD duplication and IS*5* insertion, respectively. In time–kill studies, we noted less killing against isolates with IS*5* mutations when compared with isolates with GD duplication or WT *ompK36* genotypes (Figure 6). IS*5* insertions in *ompK36* decrease *ompK36* expression and have been previously validated as a cause of elevated imipenem/relebactam MICs.^16,25^ Imipenem/relebactam MICs were less impacted by isolates that harboured a GD duplication in *ompK36*, and *in vitro* killing was similar among isolates with either WT or GD duplication genotypes. We propose the activity of imipenem/relebactam is less impacted by the constriction of porin channels mediated by GD duplication, but significantly impacted by decreased *ompK36* expression caused by IS*5* insertions. Thus, isolates harbouring a GD duplication may not demonstrate outright resistance to imipenem/relebactam unless other mechanisms, such as an increased *bla*_KPC_ copy number are present.^26,27^

By comparison, the *in vitro* activity of meropenem/vaborbactam appears to be significantly impacted by GD duplications, resulting in a 16-fold median MIC increase and less killing during time–kill experiments. Increased MICs were also identified against isolates with IS*5* genotypes. Against isolates with either GD duplication or IS*5* mutations, however, median meropenem/vaborbactam MICs were increased to 0.5 mg/L, which remains well below the CLSI susceptibility breakpoint. Based on pharmacokinetic/pharmacodynamic (PK/PD) modelling, meropenem/vaborbactam would be predicted to be effective even at the highest MICs identified in our study.^28^ This aligns with our time–kill data that showed meropenem/vaborbactam concentrations of 8 mg/L were bactericidal across *ompK36* genotypes. Moreover, the frequency of mutant selection at physiological exposures appears to be low.^29^ To date, only two cases of meropenem/vaborbactam treatment-emergent non-susceptibility have been described, and *ompK36* mutations were implicated in both.^19,26^ Like meropenem, vaborbactam accesses the periplasmic space through OmpK36,^30^ and thus both agents are reliant upon intact porin channels for cell entry.

Comparative clinical data for ceftazidime/avibactam and meropenem/vaborbactam are limited,^31^ and have not been published for imipenem/relebactam. Among patients with KPC-*Kp* infections, rates of treatment-emergent resistance are highest for ceftazidime/avibactam.^19,31–33^ Resistance to ceftazidime/avibactam is mediated by mutations in the *bla*_KPC_ Ω-loop that result in preserved *in vitro* activity against meropenem/vaborbactam and imipenem/relebactam (Table S3).^5,6,34^ Isolates resistant to meropenem/vaborbactam generally demonstrate cross-resistance to imipenem/relebactam, but not ceftazidime/avibactam.^19,26^ Interestingly, during *in vitro* selection studies with meropenem/vaborbactam, isolates with WT *ompK36* were more likely to select *ompK36* mutations than isolates with mutant *ompK36* at baseline. In the latter group, isolates were found to have an increased *bla*_KPC_ copy number following *in vitro* exposure to meropenem/vaborbactam.^29^ Similar observations have been noted for imipenem/relebactam resistance, where *bla*_KPC_ copy number and loss-of-function *ompK36* mutations are selected *in vitro*.^16^ A key difference between the agents is that mutations in *bla*_KPC_ refractory to relebactam inhibition were selected with imipenem/relebactam, but not meropenem/vaborbactam.^16,29^

The clinical impact of mutations in *ompK36* on the efficacy of ceftazidime/avibactam, imipenem/relebactam and meropenem/vaborbactam remains unclear. Animal models of *ompK36* mutant strains have demonstrated attenuated virulence,^15,35^ and porin genotypes are typically not characterized in clinical outcome-based studies.^19,32^ For now, clinicians must make decisions based on the available clinical and preclinical data. Ceftazidime/avibactam is the least impacted by *ompK36* mutations; however, selection for ceftazidime/avibactam resistance through *bla*_KPC_ mutations is a potential threat to the agent’s utility for KPC-*Kp* infections. The presence of *ompK36* mutations, particularly IS*5* insertions, elevates imipenem/relebactam MICs resulting in a proportion of isolates that are categorized as non-susceptible. In our study, simulated physiological imipenem/relebactam trough and steady-state exposures did not durably inhibit growth of KPC-*Kp* across *ompK36* genotypes. Importantly, clinical data are very limited for imipenem/relebactam when compared with ceftazidime/avibactam or meropenem/vaborbactam.^11,36^ Finally, we found that meropenem/vaborbactam MICs were elevated by GD duplication and IS*5* mutations in *ompK36*; however, median MICs were well below the current susceptibility breakpoint and within the range of optimal PK/PD target attainment.^28,29^ Taken together, our *in vitro* findings provide support for meropenem/vaborbactam as the preferred agent for treatment of KPC-*Kp* infections.

Some limitations with the current analysis should be acknowledged. First, all isolates were collected from patients at a single centre, and despite selecting strains that were genetically diverse, not all KPC-*Kp* genotypes were represented. Most notably, we did not study KPC-3-producing isolates that harboured GD duplications; however, comparable findings for KPC-3 have been reported previously.^26,27,29^ We also did not fully characterize *ompK36* mutations that were classified as ‘other’ mutations. Although ceftazidime/avibactam, imipenem/relebactam and meropenem/vaborbactam MICs were generally not impacted unless GD duplication or IS*5* mutations were present, it does not preclude the possibility that other loss-of-function mutations in *ompK36* may manifest in a similar impact on these agents. In addition, we did not investigate the *in vitro* activity against KPC variants selected by ceftazidime/avibactam treatment as part of this study, but have in prior publications.^5,6,24^ For completeness, we have summarized these data in Table S3. Secondly, our time–kill studies were limited to 24 h experiments to investigate initial killing effects, but not the presence of resistant subpopulations. In doing so, we have cautiously interpreted our findings with acknowledgement that dynamic drug exposures over longer treatment courses could yield different results. Indeed, humanized ceftazidime/avibactam exposures in a hollow-fibre infection model against KPC-*Kp* with varying genetic backgrounds may respond differently.^37^ Despite these limitations, our results shed new light on the specific impact of *ompK36* mutations on each of the recently approved β-lactam/β-lactamase inhibitor combinations. Future studies are needed to investigate comparative rates for the frequency of mutant selection, and the impact of the genetic background on treatment efficacy both *in vitro* and *in vivo*.

## Supplementary Material

dlad022_Supplementary_Data

## Supplementary data

Tables S1 to S3 are available as Supplementary data at *JAC-AMR* Online.
